# Oral Somatosensory Alterations in Head and Neck Cancer Patients—An Overview of the Evidence and Causes

**DOI:** 10.3390/cancers15030718

**Published:** 2023-01-24

**Authors:** Reisya R. Riantiningtyas, Florence Carrouel, Amandine Bruyas, Wender L.P. Bredie, Camille Kwiecien, Agnès Giboreau, Anestis Dougkas

**Affiliations:** 1 Institut Paul Bocuse Research Centre, 69130 Ecully, France; 2 Health Systemic Process (P2S), Research Unit UR4129, University Claude Bernard Lyon 1, University of Lyon, 69008 Lyon, France; 3 Section for Food Design and Consumer Behavior, Department of Food Science, Faculty of Science, University of Copenhagen, 1958 Frederiksberg C, Denmark; 4 Institute of Cancerology, Hospices Civils de Lyon, Hôpital Croix Rousse, 69004 Lyon, France; 5 Danone Nutricia Research, 3584 CT Utrecht, The Netherlands; 6 Laboratoire Centre Européen Nutrition et Santé (CENS), CarMeN, Unité INSERM 1060, Université Claude Bernard Lyon 1, 69310 Pierre-Bénite, France

**Keywords:** sensory alterations, oral somatosensory perception, nutrition, eating behaviour, cancer treatment, quality of life

## Abstract

**Simple Summary:**

The present review indicates that changes in food perception among head and neck cancer patients constitute more than changes in taste perception, but also changes in texture, temperature, and other oral sensations (e.g., spiciness, cooling sensation, carbonation). Food does not only carry physiological importance, but also conveys psychological and psychosocial values. Therefore, a comprehensive assessment of cancer patients’ food perception will allow the development of personalised dietary interventions to provide a more pleasant eating experience and improve their quality of life.

**Abstract:**

Food-related sensory alterations are prevalent among cancer patients and negatively impact their relationship with food, quality of life, and overall health outcome. In addition to taste and smell, food perception is also influenced by somatosensation comprising tactile, thermal, and chemesthetic sensations; yet studies on oral somatosensory perception of cancer patients are lacking to provide patients with tailored nutritional solutions. The present review aimed to summarise findings on the oral somatosensory perception of head and neck cancer (HNC) patients and the potential aetiologies of somatosensory alterations among this population. Subjective assessments demonstrated alterations in oral somatosensory perception such as sensitivity to certain textures, spices, and temperatures. Physiological changes in oral somatosensation have been observed through objective assessments of sensory function, showing reduced localised tactile function and thermal sensitivity. Changes in whole-mouth tactile sensation assessed using texture discrimination and stereognosis ability seem to be less evident. Available evidence indicated oral somatosensory alterations among HNC patients, which may affect their eating behaviour, but more studies with larger sample sizes and standardised assessment methods are needed. Unlike other types of cancers, sensory alterations in HNC patients are not only caused by the treatments, but also by the cancer itself, although the exact mechanism is not fully understood. Prevalent oral complications, such as xerostomia, dysphagia, mucositis, and chemosensory alterations, further modify their oral condition and food perception. Oral somatosensory perception of cancer patients is an under-investigated topic, which constitutes an important avenue for future research due to its potential significance on eating behaviour and quality of life.

## 1. Introduction

Food-related sensory alterations are common side effects of head and neck cancer (HNC) and its treatments [1,2,3]. It has adverse implications on nutritional outcomes and overall quality of life. In HNC patients, as well as in other types of cancers, sensory alterations are linked with reduced appetite and diminished food appreciation [4,5,6], altered food relationships [7,8], and changed patterns of food selection [8,9]. Sensory alterations have also been associated with reduced food intake [5,10,11], weight loss [5,10,12,13] and declined quality of life [14,15].

Taste and smell alterations are prevalent among HNC patients. Taste alterations were observed in 96% and 79% of radiated patients when assessed using subjective and objective methods, respectively [16]. Similarly, a greater prevalence of taste impairment was reflected by subjective measurements relative to objective measurements in a longitudinal study [6]. Objective assessments of taste function involve measurement of sensitivity to basic taste (sweet, sour, salty, bitter, umami) solutions, whereas subjective assessments rely on self-reported questionnaires. In the latter, “taste” is often (inaccurately) used as a colloquial term to refer to food perception as a whole, although taste only represents the five basic taste aspects of food perception [17]. The term taste is also often used to express the entire eating experience including the hedonic aspect [7,9]. On the other hand, smell alterations are underreported when measured using the subjective method (30–60%) compared to the objective method (0–100%) [18]. Therefore, the prevalent taste alterations experienced by cancer patients may not simply indicate alterations in the gustatory function, but may encompass alterations in other dimensions of food perception.

Food perception is a complex sensation which involves a cross-modal interplay between gustation, olfaction, and somatosensation. In addition to taste molecules, various receptors in the oral cavity also detect somatosensory stimuli consisting of tactile, thermal, and nociceptive stimulations. These stimuli are relayed predominantly via the trigeminal nerve to the central nervous system where they are translated into the perception of texture, temperature, and chemesthesis (e.g., irritation or burning of chilli, cooling of peppermint, tingling of carbonated drinks). Taste and smell information is integrated with oral somatosensory information in the multimodal regions of the central nervous system to form a complete picture of food perception [19,20,21]. Research on multisensory perception provided ample evidence of the importance of oral-somatosensation in overall food perception and the interrelation of somatosensory with chemosensory processing [19,22]. Hence, alterations in one of the sensory functions will modify the entire food perception and eating experience [22].

Studies on sensory alterations among cancer patients predominantly revolve around taste and smell, yet knowledge about their oral-somatosensory perception is relatively unexplored. To define strategies for alleviating food-related issues of HNC patients, there is a need for a comprehensive understanding of their food perception. Qualitative studies observed that in addition to taste and smell alterations, patients also reported somatosensory-related complaints [7,23]. However, it is unclear whether these alterations result from a perceptual origin related to the existing taste and/or smell alterations, or if there are physical and physiological changes in the somatosensory processing. The present paper aims to summarise the current evidence on the poorly understood oral somatosensory alterations in HNC patients and discuss some possible aetiologies.

## 2. Evidence of Oral Somatosensory Alterations in Head and Neck Cancer Patients

Taste and smell alterations are well-documented symptoms experienced by HNC patients [6,16,18]. However, limited studies have investigated the oral somatosensory perception of HNC patients. 

### 2.1. Subjective Perception of Oral Somatosensory Alterations in Head and Neck Cancer Patients

Descriptive studies explored the eating behaviour of HNC patients using self-reported questionnaires and interviews; these studies not only explored their taste and smell perception, but also other aspects of food perception [7]. HNC patients experienced various alterations related to oral somatosensory perception. A study on HNC survivors (*n* = 88) reported that they experienced higher sensitivity to spicy foods (27.3%), texture (27.3%), and temperature (2.3%), but 8% reported adding extra pepper and hot sauce to enhance the food [23]. The majority of the participants (77.2%) were interviewed more than a year after their treatments, yet the sensory complaints continued to persist. Similar findings were also reported in a study in which patients complained about sticky mouthfeel, difficulties in consuming food with certain textures (dry and hard food), and altered sensitivity to spices due to oral pain [7]. In a study through food-play workshops with HNC survivors, changes in chemesthetic-related experiences were also reported. Some patients developed an aversion while others developed a preference for spicy food [24]. These studies also highlighted that eating turned into an active and effortful activity due to oral pain and fear of choking, altogether leading to lowered quality of life. 

Findings on subjective perception of oral somatosensory alteration in head and neck cancer patients provided valuable insights in defining the specific problems they faced, allowing potential optimisation of their meals. However, objective assessments of their oral somatosensory functions are necessary to characterise physiological changes and the causes of these changes. Thus, psychophysical methods have been used to gain insight into the changes in tactile and thermal sensations in HNC patients (Table 1). These objective assessment methods allow quantification of the relationship between physical stimuli and the perceptions they produce [25].

### 2.2. Alteration of Tactile Functions in Head and Neck Cancer Patients

The alteration of localised oral tactile sensation in HNC patients was studied by using a point-pressure test and a two-point discrimination test. Point-pressure test measures the lowest amount of pressure that can be detected, meanwhile the two-point discrimination test measures the minimum distance at which two points of contact can be perceived as two separate points. Studies employing these methods demonstrated lower localised tactile sensations in HNC patients receiving radiotherapy [26,28] and surgery [27] relative to the control group. In studies with HNC patients with hemiglossectomy, oral sensation on intact and reconstructed tongue regions were assessed and compared with a control group. It was revealed that the intact tongue region of patients had comparable tactile sensitivity with the control group; however, a significant difference was observed between the reconstructed tongue region and the control group [30]. Similar findings were shown by Kimata et al., revealing that patients who received adjunctive radiotherapy exhibited the greatest impairment in oral tactile sensitivity [29].

In addition to the localised sensation, alteration of tactile functions was also observed using whole-mouth stimulation methods. One method is by measuring the ability to discriminate objects with different textures. HNC patients with hemiglossectomy showed a comparable texture discrimination ability with the control group [30]. Similarly, only patients with lingual nerve cuts showed impairment in texture discrimination ability [27]. Texture perception requires innervation of the whole mouth; therefore, the intact tongue region can compensate for the impaired tongue region in providing the sensory feedback. The two studies also assessed oral stereognosis ability which is the ability to perceive and recognise the form of an object in the mouth using tactile cues, in the absence of visual information. It was shown that patients had compromised oral stereognosis ability compared to the control group [30]. Meanwhile, the study by Elfring et al. showed that impaired stereognosis ability was only observed in two subgroups of patients: the group with lingual nerve intact and the group with lingual nerve cable-grafted [27]. Although oral stereognosis is also considered to be a whole-mouth stimulation method, it was suggested that the assessment predominantly relies on sensory feedback from the tip of the tongue which is impaired in patients who underwent hemiglossectomy [30].

The cross-sectional studies described above were conducted following cancer treatments; it is uncertain whether the alterations in tactile functions are caused as side effects of the treatments or if these alterations were present prior to the treatment. The longitudinal studies tend to show a trend of reduced tactile functions associated with treatments [31,32,33]. Localised tactile sensations of radiated patients were shown to decline six months after radiotherapy [31]. Similarly, stereognosis ability was not affected by cancer itself as demonstrated by the comparable performance in the shape-identification test between patient and control groups before treatments. Deteriorations were observed after radiotherapy, and further deteriorations were associated with the combined effect of surgery following radiotherapy in both oral and pharyngeal cancer groups [33]. Meanwhile, using a hole-size identification test, radiotherapy did not seem to affect stereognosis ability. Only the oral cancer group showed a decline in ability following radiotherapy and surgery [32]. It is possible that the two stereognosis methods (shape identification test and hole size identification test) may innervate different types or regions of mechanoreceptors and involve different mechanisms of tactile perception.

### 2.3. Alterations of Thermal Sensitivity in Head and Neck Cancer Patients

Altered thermal sensitivity was demonstrated by lower hot/cold discrimination ability in HNC patients. The hot/cold discrimination test measures one’s ability to differentiate between hot and cold stimuli using various tools (e.g., dental mirrors, metal rolls, or cotton swabs submerged in two different temperatures of water). Impaired thermal sensitivity was observed in HNC patients receiving surgical treatments [27]. The intact tongue region of patients had comparable thermal sensitivity with the control group; however, a significant difference was observed between the reconstructed tongue region and the control group [30]. The two studies showed indications that the impaired thermal sensitivity was associated with surgical procedure; however, since the studies are cross-sectional, it is not possible to confirm if the impairment occurred following the surgery or if it was already present before the surgery. In a longitudinal study, it was demonstrated that thermal sensitivity remained unchanged after radiotherapy, but deteriorated six months after surgery following radiotherapy [31]. It could not be confirmed whether the impairment was due to the surgery itself or if it was a cumulative effect of radiotherapy and surgery. 

Taken together, altered somatosensory perception is caused not only by perceptual changes, but also by physiological changes, as demonstrated by psychophysical studies. The studies showed altered thermal sensitivity and localised tactile functions, but findings for the whole-mouth tactile sensation (texture discrimination, stereognosis) are less conclusive. Altered sensitivity to chemesthetic sensations, such as spiciness (capsaicin), is not uncommon among HNC patients, but psychophysical studies using chemesthetic stimuli among cancer patients have not been reported. Therefore, it is not possible to ascertain whether the possible alterations in chemesthetic perception are attributed to physiological changes or merely a result of perceptual changes. More studies with larger sample sizes and standard assessment methods are needed to estimate the prevalence and the severity of somatosensory alterations. Studies associated the alterations with treatments rather than with the cancer itself, yet more longitudinal studies are needed to specify when exactly (and for how long) these alterations occur.

## 3. Aetiology of Oral Somatosensory Alterations in Head and Neck Cancer

To understand the causes of oral somatosensory alterations in HNC, it is necessary to understand the physiology of sensory perception. The mechanism can be conceptually divided into three stages: stimulation, transduction, and interpretation. First, food (i.e., stimulus) that enters the mouth stimulates various sensory receptors. The stimulus is transduced into action potentials by ion-gated channels and conveyed to the central nervous system via the nerves. Finally, this leads to an encoding process of interpreting the action potential into perception which also involves integration of the different sensory modalities in the multimodal regions of the central nervous system [34]. Altered somatosensory perception may occur if any of these processes are disrupted. Although the pathophysiology of oral somatosensory alterations is poorly understood, the following section outlines the evidence for a range of possible aetiologies. Table 2 summarises the physical and physiological changes associated with cancer and its treatments on somatosensory perception. 

### 3.1. Impact of Tumour and Cancer Inflammation on Oral Somatosensation in Head and Neck Cancer Patients

The disease itself may alter oral somatosensory perception of HNC patients. As described in Section 2.2, the decline in tactile sensitivity appears to be associated with the treatments rather than the disease states, but there are possible mechanisms that the cancer itself may alter sensitivity to noxious stimuli. It can be a result of anatomical changes where the primary tumour compresses surrounding nerves responsible for conveying sensory information, or it damages the mucosal lining composed of sensory receptors [35]. It may also involve molecular changes, such as the activation of toll-like receptors and interferon during inflammation, which may disrupt normal regeneration of sensory receptor cells [36]. 

Mechanistic studies using animal models have shown that HNC causes nociceptive and thermal sensitisation through various mechanisms. Oral squamous cell carcinoma can sensitise peripheral trigeminal nerve terminals via increased spontaneous firing of lingual fibres, which may result in altered sensitivity [37]. Oral cancer may also alter sensitivity to capsaicin and noxious heat due to overexpression of protease-activated receptor-2, tumour necrosis factor-alpha, nerve growth factor, and adenosine triphosphate, which can sensitise transient receptor potential vanilloid type 1 (TRPV1), an ion channel responsible for the transduction of capsaicin and noxious heat [38]. Cancer cells also release a variety of pain mediators, such as bradykinin, cytokines, chemokines, nerve growth factor, prostaglandins, and several vascular factors that can activate and sensitise nociceptive primary afferents, resulting in intense and spontaneous pain commonly experienced by HNC patients [39,40,41]. These mechanistic studies provided indications of physiological changes that can contribute to altered chemesthetic sensitivity; however, there is insufficient published evidence to confirm this as there are no psychophysical studies on chemesthetic sensitivity in HNC patients. 

### 3.2. Impact of Cancer Treatments on Oral Somatosensation in Head and Neck Cancer Patients

In other types of cancer, it is suggested that the cancer in itself does not cause sensory alterations, but rather the cancer treatments are the cause [17]. The treatments for HNC are surgery, radiotherapy, and chemotherapy. Each treatment has potential impacts on oral somatosensory perception of HNC patients through different mechanisms.

#### 3.2.1. Impact of Surgery on Oral Somatosensation in Head and Neck Cancer Patients

Surgery can result in anatomical changes. Removal of cancerous tumours may include removal of key organs necessary for sensory perception, such as partial or total removal of the tongue. If the surgery requires major tissue removal, such as jaw, skin, pharynx, or tongue, reconstructive surgery may be done to replace the missing tissue; however, it does not guarantee total restoration of oral sensations. Reduced localised tactile and thermal sensitivity have been observed following reconstructive surgery on the oral cavity or pharynx [27,29,30,31,32,33]. This can be attributed to nerve impairment following the surgery, as patients who underwent reconstructive surgery with innervated flaps displayed greater sensory recovery compared with patients who received the non-innervated flaps [29]. Similarly, patients with a cut lingual nerve demonstrated significantly lower tactile and thermal sensitivity, while those with an intact lingual nerve displayed comparable sensitivity to the control group [27,31,32]. However, the texture discrimination ability, which requires whole mouth perception, was not affected [27,30].

Bartoshuk et al. [42] summarised that anaesthesia or mild damage to chorda tympani resulted in elevated whole-mouth sensitivity, both taste and somatosensation. This phenomenon is known as the release of inhibition or disinhibitory effect model, which proposes that in normal conditions, the two sensory nerves (glossopharyngeal nerve and lingual nerve via chorda tympani) partially and mutually suppress one another. In the event of anaesthesia or mild damage to one of the nerves, the central suppression will be eliminated leading to a heightened sensitivity on the intact nerve, whereas extensive damage or damage to both nerves leads to diminished perception [42].

#### 3.2.2. Impact of Radiotherapy on Oral Somatosensation in Head and Neck Cancer Patients

Cancer patients undergoing radiotherapy on the head and neck region may experience acute and chronic changes to their soft tissue, as well as transient and permanent sensory alterations. HNC patients receiving radiotherapy showed lower oral tactile sensitivity compared to healthy controls [26,28]; however, the mechanism is unclear. A possible explanation is a reduction in the number of fungiform papillae which contain sensory receptors [43]. In a longitudinal study, HNC patients showed reduced taste sensitivity five weeks after radiotherapy, with improvement by the 11th week [44]. The study also explored the underlying physiological mechanism using an animal model and showed that radiotherapy not only damaged the taste buds, but also altered the papillae thickness. Another study lends credence to the theory of papillae damage as a pertinent factor in taste alterations following radiotherapy. It was found that the fungiform papillae of HNC patients with taste disorders had thicker epithelia compared with healthy subjects [45]. As fungiform papillae are also responsible for thermal and tactile perception [46], it is likely that the damage will also influence oral somatosensory perception. Oral somatosensory decline can also be caused by radiotherapy-induced damage of mucosal sensory receptors located within the oral cavity and oropharynx [28].

#### 3.2.3. Impact of Chemotherapy on Oral Somatosensation in Head and Neck Cancer Patients

Studies investigating the effect of chemotherapy on oral somatosensory alterations among HNC patients have not been reported, since it is uncommon for HNC patients to receive chemotherapy as the only treatment. Findings on the effect of chemotherapy on taste and smell alterations in various types of cancers are inconsistent, and there is insufficient evidence to conclude whether the taste and/or smell changes following chemotherapy are attributed to perceptual or physiological changes [12,47].

A popular hypothesis on the effect of chemotherapy on various cancers is associated with the cytotoxic agents, such as cisplatin and doxorubicin. These cytotoxic agents target rapidly dividing cancer cells by disrupting their proliferating activity, but may also affect other rapidly dividing cells including sensory receptor cells [35,48]. There is also evidence of TRPV1 sensitisation following chemotherapy, resulting in heightened sensitivity to capsaicin [49]. Another proposed mechanism is the inhibition of the hedgehog pathway, which regulates and restores taste buds [50]. Inhibiting the hedgehog pathway using cancer drugs was shown to cause loss of taste buds, and consequently eliminating the relay of taste information to sensory nerves. It was expected that the inhibition will also affect somatosensory perception; however, it was shown that touch and temperature responses stayed intact [51]. This is because the hedgehog signal blocking only eliminates taste buds, but leaves the soma and nerve fibres intact [52]. 

### 3.3. Secondary Effects and Consequences of Treatment That Impact Oral Somatosensation in Head and Neck Cancer Patients

There are several secondary effects and consequences that accompany cancer treatments. Some of these effects directly influence oral conditions and may impact the oral somatosensory perception. Xerostomia, dysphagia, mucositis, and chemosensory alterations influence different aspects of somatosensory perception. 

#### 3.3.1. Impact of Xerostomia on Oral Somatosensation in Head and Neck Cancer Patients

Xerostomia, which refers to the perception of dry mouth, is a prevalent adverse effect reported by HNC patients. Damage to the salivary glands or the blood vessels and nerves supplying the glands causes a reduction in salivary flow. It usually occurs as a side effect of radiation and translates into sensations of dry mouth and thickened or stringy saliva [53,54]. It was reported that 64% of long-term HNC survivors and 91.7% of HNC patients receiving concurrent chemoradiotherapy experienced xerostomia [55,56]. Around 60% of patients developed xerostomia during radiotherapy, which remained even after two years [57]. Xerostomia is often accompanied by taste alterations, difficulty in speaking, increased risk of caries, oral pain, and burning sensation [53]. Patients with xerostomia are also more likely to experience swallowing difficulties, food sticking in the mouth and/or throat, needing a water assist when swallowing, and a change in taste, which affects their overall sensory perception and food enjoyment [54]. Textural and mouthfeel sensations, such as viscosity, stickiness, creaminess, and astringency, were shown to be influenced by the amount, composition, and buffering capacity of saliva [58]. Therefore, it is expected that xerostomia will influence texture and mouthfeel perception of HNC patients. 

#### 3.3.2. Impact of Dysphagia on Oral Somatosensation in Head and Neck Cancer Patients

HNC patients reported dysphagia or swallowing difficulty among the adverse effects. Dysphagia is a multifactorial condition which can be caused as a side effect of surgery and other cancer treatments [59]. Cancer in the head and neck region may result in anatomical changes including reduction of pharyngeal or hypopharyngeal space, incontinence of oral cavity, and alteration of pharyngeal peristalsis. These collectively result in impaired swallowing function [60]. In addition, radiotherapy on the region was also shown to cause dysphagia. A longitudinal study reported that at the onset of the radiotherapy, 18% of patients developed dysphagia; this number increased to ~90% in the fifth week, and persisted after two years for ~20% of the patients [57]. Another study reported that 78.7% of HNC patients receiving concurrent chemoradiotherapy developed dysphagia [55]. Dysphagia was associated with difficulties in eating certain food consistencies and difficulties in managing dry food; therefore a lot of these patients required texture-modified diets [59].

#### 3.3.3. Impact of Mucositis on Oral Somatosensation in Head and Neck Cancer Patients

Mucositis occurs in the majority of patients receiving radiotherapy on the oral and oropharyngeal mucosa. Mucositis is the lesion of oral mucosa specifically associated with cytotoxic cancer therapy [61]. In its mild form, mucositis presents as mucosal erythema accompanied by a burning sensation, but in its advanced stage, it presents as deep and painful ulcerations of oral mucosa. In one study, it started to develop two weeks following radiotherapy and by the fifth week, almost 90% of the patients had mucositis; this persisted for two years in 60% of the patients [57]. It could also be a side effect of chemotherapy via the proinflammatory cytokines [61]. Up to 74% of HNC patients receiving concurrent chemoradiotherapy developed mucositis [55]. Patients with mucositis are more likely to be sensitive to spicy food and hot temperatures [62].

#### 3.3.4. Impact of Chemosensory Alterations and Other Oral Complications on Oral Somatosensation in Head and Neck Cancer Patients

Chemosensory alterations are common side effects experienced by HNC patients [16,18]. Taste and/or smell alterations may contribute to oral somatosensory alteration through the interrelation between the three modalities in the multimodal region of the central nervous system [40]. A study showed that individuals with taste dysfunction also displayed lower tactile acuity, suggesting a possible alteration in their texture perception [63]. Other possible oral complications that may alter patients’ somatosensory perception are summarised in Table 3.

Oral-somatosensory alterations in HNC patients are caused by the cancer itself and the various cancer treatments. Physical and physiological changes influence different levels of sensory processing. In relation to the secondary effects of cancer treatments, oral complications cause the oral cavity to be more sensitive to spices, noxious temperatures, and certain textures (e.g., dry and hard textures), consequently, limiting their food choices and food intake. To address these changes, it is beneficial to constantly monitor the oral hygiene of patients to prevent worsening oral conditions, provide saliva replacement or stimulants, and modify the sensory properties of meals based on their perception. 

## 4. Conclusions and Future Perspectives

The current review focused on investigating oral somatosensory perceptions of HNC patients in relation to food perception. Causal relationships have not been established between sensory alterations and nutritional outcomes. However, mounting evidence has shown associations between sensory alterations on food perception and altered food intake. Sensory alterations, particularly taste and smell, have been associated with weight loss, reduced food intake, and diminished quality of life. HNC patients experience altered sensitivity to certain food textures, spices, and temperatures, but the experience varies between individuals. Objective assessments indicated reduced localised tactile function and thermal sensitivity, but findings on chemesthetic sensitivity and whole-mouth sensation are less conclusive. Collectively, it seems evident that altered food perception does not only constitute taste and smell aspects, but also the oral somatosensory aspect. However, more studies with larger sample sizes and standardised assessment methods are needed to estimate the prevalence of oral somatosensory alterations among HNC patients. 

In the case of HNC, the cancer site may influence oral somatosensory perception through physiological changes due to its location directly involved in food intake. Mechanistic studies with murine models show indications that HNC triggers nociceptive sensitisation through various pathways. Additionally, cancer treatments influence sensory perception, including oral somatosensory perception, through disruption to the receptors and nerve impairment, but the exact mechanism is not fully understood. Oral complications following the disease and treatments, such as xerostomia, dysphagia, mucositis, and chemosensory alterations, result in altered oral conditions and perceptual changes which further influence patients’ food perception.

Disorders of food perception are generally difficult to diagnose and treat as food perception is not only influenced by the physiological state of sensory systems, but also by the perceptual and hedonic aspects. Adding to the challenge, the multidimensionality of sensory perception makes it difficult to disentangle an eating experience into individual sensory modalities, yet the distinction is essential to address specific interventions. In addition to the aforementioned knowledge gaps, critical questions such as: the duration and severity of oral somatosensory alteration, its correlation with taste and smell alterations, as well as its significance on eating behaviour, remain to be investigated. Without enough knowledge in this area, there is a limited basis for developing appropriate assessment frameworks or potential interventions. The present review brings attention to the need for a multidisciplinary perspective, as food perception is one of the key drivers affecting eating behaviour. Food does not only carry physiological importance, but also conveys psychological and psychosocial values. Therefore, a comprehensive assessment of cancer patients’ food perception will allow the development of personalised dietary interventions to provide a more pleasant eating experience and improve their quality of life. 

## Figures and Tables

**Table 1:** Summary of psychophysical studies investigating oral somatosensory perception in head and neck cancer patients.

Reference	Population	Tumour Subsite, Treatment	Sensory Tests	Findings
Aviv et al. 1992 [26]	HNC patients 12–36 mo after RT (*n* = 20), control (*n* = 90). Cross-sectional	Tonsil or nasopharynx, RT	- Two-point discrimination (tongue and floor of mouth)	Radiated HNC patients were less sensitive in oral tactile acuity test than the control group
Elfring et al. 2012 [27]	HNC patients (*n* = 30), control (*n* = 30). Cross-sectional	Tongue, surgery with or without RT or CT	- Point pressure - Two-point discrimination - Hot/cold discrimination with dental mirrors 55 and 3 °C - Texture discrimination of resin - Stereognosis	All patients with lingual nerve disruption exhibited significantly poorer outcomes in the point pressure test, 2-point discrimination test, and hot/cold discrimination test. No difference in texture discrimination, less conclusive for oral stereognosis
Bearelly et al. 2017 [28]	HNC patients (*n* = 34), control (*n* = 23). Cross-sectional	Oral cavity and oropharynx, RT	- Point pressure (tongue, buccal mucosa, soft palate)	Elevated sensory threshold in patients compared to the control group
Kimata et al. 1999 [29]	HNC patients who received innervated (*n* = 15) and noninnervated (*n* = 13) free flaps reconstruction surgery. Cross-sectional	Tongue, hemiglossectomy	- Point-pressure - Two-point discrimination - Hot/cold discrimination with cotton swabs	Sensory recovery was significantly better with innervated thigh flaps than non-innervated ones for all sensory modalities and better with innervated abdominis flaps than non-innervated ones for all modalities except thermal sensitivity.
Loewen et al. 2010 [30]	HNC patients with innervated free flap reconstruction surgery (*n* = 8), control (*n* = 8). Cross-sectional	Tongue, hemiglossectomy	- Point-pressure test - Two-point discrimination - Hot/cold discrimination with dental mirrors - Texture discrimination with acrylic resin	Sensation of intact tongue tissue after reconstruction of the hemitongue did not differ from controls. Although some sensory ability was restored to patients’ reconstructed tongue, differences existed between the patient group and controls. However, the texture discrimination ability was comparable with controls.
Bodin et al. 2004 [31]	HNC patients (*n* = 27), control (*n* = 20). Longitudinal (diagnosis, post-RT, 6 mo post-surgery, 12 mo post-surgery)	Oral or pharyngeal, RT and surgery	- Point pressure test - Hot/cold discrimination with metal rolls 44 and 28 °C	Deterioration of tactile and thermal sensitivity at 6 mo after surgery
Bodin et al. 1999 [32]	HNC patients (*n* = 31), control (*n* = 20). Longitudinal (before RT, after RT, 6 mo after surgery following RT, 12 mo after surgery following RT)	Pharyngeal and oral cavity, surgery following RT	Stereognosis: hole size identification	The oral group did not show a decline in the hole-size identification ability after radiotherapy, but did 6 mo after the surgery following RT. Deterioration was persistent for 1 year after. The pharyngeal group did not change performance in hole-size identification at any time point.
Bodin et al. 2000 [33]	HNC patients (*n* = 30), control (*n* = 20). Longitudinal (before RT, after RT, 6 mo after surgery following RT, 12 mo after surgery following RT)	Pharyngeal and oral cavity, surgery following RT	Stereognosis: shape identification	The mere existence of a tumour did not affect shape-recognition abilities. RT caused some impairment of shape recognition, while the combined effect of surgery following RT caused significant deterioration. No effect of tumour location was observed.

CT: chemotherapy; HNC: head and neck cancer; RT: radiotherapy.

**Table 2:** Overview of mechanisms in which cancer and its treatments alter oral somatosensation.

Mechanisms	Peripheral Level	Nerve Level
Cancerous Cells	Disruption of regeneration of sensory receptor cells Over-expression and sensitization of ion channels (e.g., TRPV1)	-
Tumour	Disruption of mucosal receptors on sites of tumour growth	Compression of sensory nerve on sites of tumour growth
Surgery	-	Impairment on sensory nerve
Radiotherapy	Damage to papillae and mucosal sensory receptors	-
Chemotherapy	Disruption of regeneration of sensory receptors	-

TRPV1: Transient receptor potential vanilloid type 1.

**Table 3:** Summary of other oral complications influencing oral somatosensory perception.

Oral Manifestation	Description	Influence on Oral Perception
Osteoradionecrosis [1,64]	Condition of bone and mucosal breakdown after RT. Incidence rate is 4–37% in HNC patients. Mandible surgery and tooth extraction before RT, tobacco use, and treatment dose were associated with the development of ORN	Chronic oral pain and irritation
Temporomandibular disorder [65]	A collective term to describe masticatory pain and dysfunction. A study showed that 68, 94, and 81% of HNC patients had TMD before, 6 mo after treatments, and 12 mo after treatments, respectively	Difficulty with certain textures, oral pain, and discomfort
Trismus [66,67,68]	Restricted mouth opening caused by radiation damage on the temporomandibular joint, resulting in scarring and fibrosis of pterygoid muscles and ligaments. Prevalence among HNC patients ranges from 5–86% depending on tumour location, treatment, and stage of treatment	Oral discomfort, difficulty chewing and swallowing certain food textures
Trigeminal Neuralgia [69]	A chronic syndrome is signified by recurrent facial pain in the dermatome of the trigeminal nerve (fifth cranial nerve). It is associated with nerve injury or lesion.	Heightened sensitivity to temperature and trigeminal sensation
Burning mouth syndrome [70]	Constant burning sensation or discomfort. Multifactor, unclear aetiology may be caused by a damaged chorda tympani, nerve-stimulation phantoms	Intensified trigeminal sensations, sensitivity to hot temperature
Opportunistic infection (e.g., oral candidiasis) [1,71]	Infection caused by fungi, bacterial, or viral due to disrupted homeostasis (RT, mucositis, hyposalivation) leading to dental caries (>25% of patients receiving RT)	Mucosal pain, dysphagia, taste change, trigeminal sensitivity, sensation of coating in the mouth
Periodontal disease [1]	Loss of tooth-supporting tissue and alveolar bones. Oral manifestation of RT through mucositis and changes in oral microbiome	Pain and infection in jaw bones, tooth loss, reduced sensitivity to particles (texture), chewing difficulty

ORN: Osteoradionecrosis; TMD: Temporomandibular disorder.
